# Cystic Fibrosis Lung Infections: Polymicrobial, Complex, and Hard to Treat

**DOI:** 10.1371/journal.ppat.1005258

**Published:** 2015-12-31

**Authors:** Laura M. Filkins, George A. O’Toole

**Affiliations:** Department of Microbiology and Immunology, Geisel School of Medicine at Dartmouth, Hanover, New Hampshire, United States of America; McGill University, CANADA

## It’s Not Just about *Haemophilus influenzae*, *Staphylococcus aureus*, and *Pseudomonas aeruginosa*

**Fig 1:**
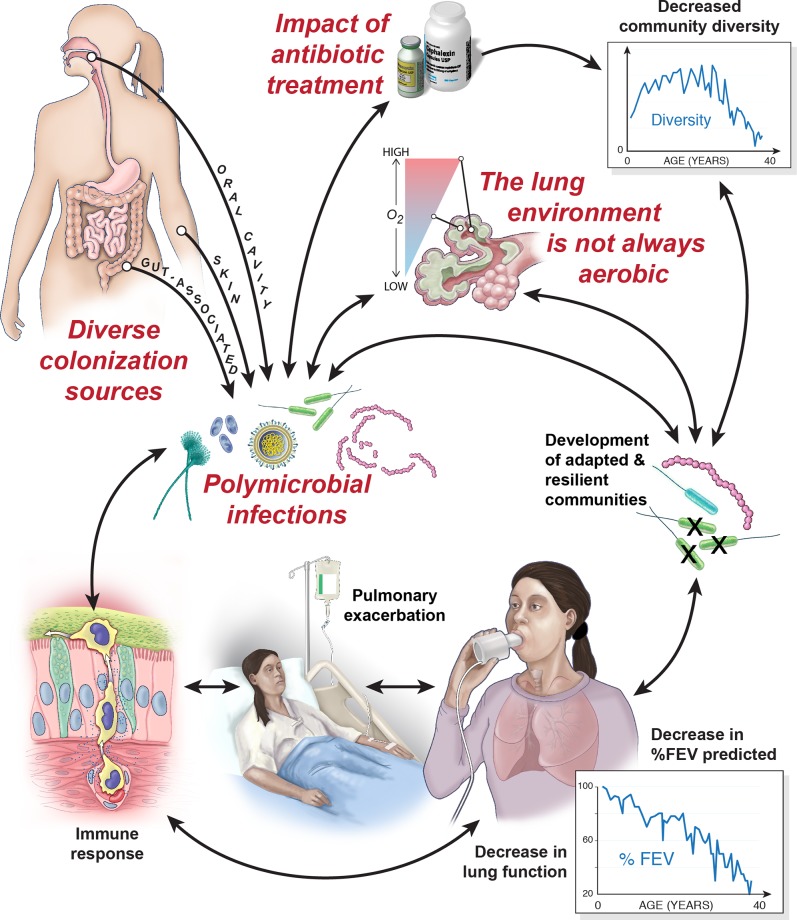
Cystic fibrosis lung infections are polymicrobial, complex, and challenging to treat. The airways of patients with CF are colonized from various host (as indicated) and environmental (not depicted) sources. Patients subsequently develop chronic, polymicrobial infections composed of diverse bacterial, fungal, and viral organisms. These polymicrobial communities both influence and are impacted by their human host through complex, multifactorial interactions. As highlighted in the figure above and throughout this review, CF lung microbial communities encounter frequent antibiotic therapy, host immune factors, and an altered lung environment (including the presence of hypoxic [low oxygen] and anoxic [no oxygen] regions) throughout disease progression, all of which contribute to the development of chronic communities that often have decreased microbial diversity and are populated by organisms that have become highly adapted and resilient to treatment. The combination of diverse colonization sources, dynamic inter-domain interactions, microbial adaptation, environmental factors, and patient therapy mediates patient outcome. Eventually, chronic pulmonary infection culminates in a decline in lung function, which becomes most severe during pulmonary exacerbations and late stage disease progression. This steep decline in lung function ultimately leads to respiratory failure, the primary cause of morbidity and mortality in CF patients today [6]. Figure illustration and design copyright 2015 William Scavone and used with permission.

Historically, *H*. *influenzae* and *S*. *aureus* were considered to be the primary organisms infecting the airways of infants and children with CF, followed by *P*. *aeruginosa* or *Burkholderia cepacia* complex during adulthood. These bacteria continue to play important roles in CF respiratory infections and clinical outcome; however, with the advent of improved culture methods and culture-independent approaches, including deep sequencing technology, it is clear that the airways of patients with CF are chronically colonized with complex, polymicrobial infections. In 2003, Rogers and colleagues revolutionized our understanding of CF lung infections through their identification of complex bacterial communities in sputum and bronchoscopy samples using a culture-independent, molecular-based approach, terminal restriction fragment length polymorphism profiling. This study was the first to recognize the role of highly prevalent and diverse bacterial species previously unrecognized in CF lung infections, including obligate anaerobes [1]. Since then, several additional studies utilizing culture-independent approaches have confirmed that the airways of patients with CF are chronically colonized with diverse bacterial, fungal, and viral taxa [2–5]. These polymicrobial communities are highly individualized to each patient and promote intricate inter-microbial and host—pathogen interactions, which alter the lung environment, impact response to treatment, and direct the course of disease (summarized in Fig 1).

Core genera, including *Streptococcus*, *Pseudomonas*, *Prevotella*, *Veillonella*, *Neisseria*, *Porphorymonas*, and *Catonella* are detected in abundance in the majority of adult sputum samples [7]. In addition to this core, deep sequencing typically identifies 50–200 unique operational taxonomic units in a single CF respiratory sample. Furthermore, the advancement of molecular identification approaches has greatly enhanced our recognition of the diverse fungi (including *Candida* spp., *Malassezia* spp., and *Aspergillus* spp.) and viruses (including influenza and respiratory syncytial virus) that co-inhabit the lungs of patients with CF [2,4,8].

Accompanying the explosion of organisms recognized in these multi-domain communities comes the task of determining the role of such microbes in CF lung disease. In the recently accepted context of highly individualized, complex, polymicrobial communities, answering the question of “who’s the pathogen?” has become a non-trivial challenge of both clinical diagnostic and academic research focus. Emerging pathogens (microbes that directly contribute to disease progression and poor patient outcome) of interest are bacterial (*Streptococcus* milleri group spp., nontuberculous mycobacterium), fungal (*Trichosporon* spp.), and viral (rhinovirus). Meanwhile, other organisms previously considered to be pathogenic in the context of CF lung infections are now more widely thought of as normal microbiota and unlikely pathogens, including *Stenotrophomonas maltophilia*, *Achromobacter* spp., *Ralstonia* spp., *Burkholderia gladioli*, and *Streptococcus pneumoniae* [8].

Defining individual organisms as pathogenic (or not) is a convenient tool for standardizing treatment approaches. However, as we now understand the complexity of these polymicrobial infections, it is critical to begin interpreting the function and impact of infecting microbes in the context of their community, not as individuals. To this point, Sibley and colleagues demonstrated that co-infection of oropharyngeal microbes with *P*. *aeruginosa* frequently altered (strain-dependent increase or decrease) host survival in a *Drosophila* model, compared to mono-infection of either organism [9].

Furthermore, distinguishing between carriage (not contributing directly to disease) of microbes versus their role as bona fide pathogens is often unclear. For example, rhinovirus detection in the upper respiratory tract of infants and children is common and is thought to typically have minimal impact on the respiratory disease course in this young cohort [10]. However, increased detection of rhinovirus (as well as influenzae A and B viruses) was also reported during pulmonary exacerbation, compared to periods of stability [11]. A similar dual role of carriage and pathogenic potential has been described for emerging bacterial pathogens of the *Streptococcus* milleri group and the fungus *Candida albicans* [8,12–14]. Understanding the environmental, host, or inter-microbial triggers that lead to the pathogenic role for these and other microbes is imperative for proactive patient treatment and prevention of disease worsening.

## The Lung Environment Is Not Always Aerobic

Contrary to common intuition, the lungs are not entirely aerobic—especially the airways of patients with CF. In CF, mutations in the cystic fibrosis transmembrane conductance regulator gene lead to decreased chloride ion secretion and dehydration of the airway surface liquid layer, which leads to deficient mucociliary clearance and development of a thick mucus layer. The combination of thickened mucus and decreased clearance further facilitates the formation of mucus plugs that can obstruct the airways and form a protected niche for microbes [15]. Within this thick mucus, and particularly within the plugs, a steep oxygen gradient forms with hypoxic (low oxygen) or anoxic (no oxygen) regions (see Fig 1). Additionally, mucus hypoxia or anoxia may be further enhanced by oxygen consumption and growth of colonizing organisms, such as *P*. *aeruginosa* [16]. The airways of patients with CF are heterogeneous in regard to the tissue environments (e.g., localized regions of high versus low oxygen and regional variation in inflammation) and microbial communities (e.g., the abundance of microbes and composition of communities in different regions of the airway) [17]. This heterogeneity impacts localized host and microbial interactions and, ultimately, disease progression. Microbes equipped to survive under diverse host conditions, in particular low or varied oxygen concentrations, may have increased potential to chronically colonize the airways and impact patient outcome. Species traditionally thought of as aerobic microbes are often also equipped to survive and grow in low- or no-oxygen environments. *P*. *aeruginosa*, for example, can grow by respiring nitrate and nitrite in anoxic environments and has multiple terminal oxidases that support aerobic respiration in low oxygen conditions [18]. *P*. *aeruginosa* also has the capability to ferment arginine and pyruvate to provide maintenance energy [19]. Furthermore, the viscous mucous and low-oxygen environments may be advantageous for some species, such as *P*. *aeruginosa*, that experience enhanced antibiotic tolerance under such conditions [19].

Finally, the high prevalence and abundance of anaerobic species in the CF airway provides empirical evidence for anoxic niches within the lung [1]. These anaerobes are not merely dead cells transiently inhaled into the lungs; patient lung samples house viable, diverse anaerobic species that can be recovered in culture [20], and longitudinal microbiome studies have further shown that these anaerobes are chronic residents [5]. The role of obligate anaerobes and non-oxygen respiring microbial lifestyles in the development of chronic polymicrobial communities and disease progression is currently not well understood and is a growing focus in the field.

## Who Did It? Lung Infections Are Seeded by Our Mouth, Nose, Skin, Gut, Siblings, Neighbors, and Dirt

In the absence of predominating *P*. *aeruginosa*, oral-associated microbes can be the most prevalent and abundant species found in the CF lung. Indeed, microbiome studies indicate that *P*. *aeruginosa* may only be predominant in approximately 50% of adult patients [13]. Importantly, the oral cavity plays a significant role in seeding the lower respiratory tract with diverse microbes including *Streptococcus* spp., *Prevotella* spp., *Veillonella* spp., *Fusobacterium* spp., *Rothia* spp., and others (see Fig 1) [5,7,13]. Despite similar origins, the community structure of lung samples and corresponding mouth wash samples are distinct, indicating that the lung environment is unique from the upper respiratory tract and selects for a separate community [21].

It is important to note that oral-associated microbes are detected in the airways of individuals with CF upon 16S rRNA gene deep sequence analysis of bronchoscopy-guided protected brush samples (D.A. Hogan and A. Ashare, personal communication), which physically separate the sample from contamination by the upper airways and oral cavity during sampling. Thus, although it is possible, and even likely, that many lower airway samples (including sputum) are partially contaminated by microbes in saliva during passage through the oral cavity, oral-associated microbes are clearly prominent residents in the lower respiratory tract of patients with CF.

Other body sites also play a role in the development of the microbial communities of CF airways (see Fig 1). Nasal- and skin-associated microbes (such as *S*. *aureus* and *Corynebacterium* spp.) can colonize early in life and, in some patients, persist through adulthood [3,5]. Additionally, particularly in infants and young children, gut colonization with specific genera (including *Escherichia*, *Enterococcus*, and others) may precede colonization in the airways, either via direct or indirect interactions with the airways [3]. Furthermore, gut microbiota are central to training the immune system in young children—an event that is described as later impacting immune modulation in the lungs [22] and, thus, may guide which species successfully colonize and persist in the CF airway.

For some species, patient-to-patient transmission and the environment are primary sources of infection, rather than the patient’s own microbiota. The common adulthood pathogens *P*. *aeruginosa* and *B*. *cepacia* complex are typical soil bacteria and are most frequently acquired from environmental reservoirs [8]. However, some strains of *Burkholderia* spp., as well as nontuberculous mycobacterium, may also be spread among patients, potentially to the extent of becoming epidemic strains within the CF patient population—a particular concern for infection control [8]. Exemplary of these environmental and patient reservoirs, the lung microbiota of co-inhabiting pediatric siblings is more similar than patients living separately [23].

## The Impact of Antibiotic Treatment on Community Composition and Patient Outcome Is Complex and Not Well Understood

Throughout their lives, patients with CF receive antibiotic treatment both intermittently, for chronic infection management, and aggressively, during hospitalization for pulmonary exacerbation. Despite this long-term exposure to a range of antimicrobial agents, microbiota of the CF lung are not cleared, as would be expected with other common bacterial infections. Viable bacterial cell counts generally only fall approximately 10-fold in sputum after antibiotic treatment for pulmonary exacerbation [20], and the impact of antibiotics on total airway bacterial populations is unknown. Molecular analyses of viable bacterial populations reveal decreased microbial diversity within 72 hours of initiating treatment [24]; however, the impact of antibiotic treatment is transient, and baseline communities generally recover within 30 days [5].

Not surprisingly, bacteria acquire multi-drug resistance, and microbial biology and CF lung physiology may further contribute to antibiotic tolerance. Low oxygen environments or growth in a biofilm may enhance antibiotic tolerance [19]. This multifactorial contribution toward high tolerance to antibiotics may in part explain the persistence of microbial communities in the airways of patients with CF despite decades of ongoing antibiotic therapy.

Despite our limited understanding of the mechanism of action, patients treated with combination antibiotics during hospitalization report “feeling better” and show signs of clinical improvement and increased lung function. Paradoxically, antibiotic treatment results in decreased microbial diversity short-term, a trait usually associated with decreased health [5,24]. In adults, decreased diversity over time correlates with progressive lung disease and decreased lung function [5,25]. While clinical correlations have been observed between decreased microbial diversity and poorer patient status, the causative factor(s) (such as absence of a specific beneficial microbe, multiple beneficial microbes, a key microbial function carried out by a subset or community of individuals, the host response to such communities, or others) of this phenomenon are not known. Methods of promoting microbial diversity in the airways have been proposed, ranging from decreased use of antibiotics to the enhanced use of prebiotics and probiotics; however, these approaches remain largely unexplored through clinical trials. Ultimately, the optimal balance between aggressively treating patients during pulmonary exacerbation and supporting clinical stability through maintenance therapy (both of which undoubtedly contribute to extended patient life span) versus minimizing the decline in microbial diversity remains a topic for debate.

## The More We Learn, the Less We Understand about Why Patients Get Sick (and What Makes Them Better)

Throughout life, patients experience periodic pulmonary exacerbations characterized as flares of decreased lung function, increased cough and inflammation, chest pain, and often weight loss. Historically, it was speculated that pathogen blooms or increased total bacterial load are responsible for triggering these acute episodes. However, several recent independent studies have demonstrated that overall bacterial abundance, community composition, and diversity are largely unchanged when comparing paired samples from patients during clinical stability and onset of exacerbation (before antibiotic treatment; [5] and others). Furthermore, neither total viable cell counts nor *P*. *aeruginosa* viable counts are consistently altered during an exacerbation [20].

Despite broad measures of community dynamics often remaining unchanged in patients treated with antibiotics, changes in immune response, such as increased inflammation, suggest altered host—pathogen interactions resulting from biological changes we may be missing. For example, the increased abundance of *Streptococcus* milleri group, until recently not associated with CF exacerbation, may contribute to pulmonary exacerbation [14]. Changes in unlikely pathogens, such as increased abundance of *Gemella* spp., may serve as biomarkers of clinical worsening [26]. Additionally, viruses, especially influenza A, have been correlated with more severe symptoms during an exacerbation [2,8].

Alternatively, while the community structure may not change during exacerbation, biological functions might; changes in microbial gene expression, virulence, or metabolite production may alter disease course. For example, in a study by Twomey and colleagues, enhanced accumulation of metabolites associated with anaerobic metabolism, including lactate and putrescine, were observed to correlate with the exacerbation state [27]. Overall, our current understanding of functional community changes during states of stability versus disease is limited. However, the development of new methodologies and affordable, high-throughput technologies in the areas of metagenomics, metatranscriptomics, and metabolomics is quickly providing the tools needed to investigate these outstanding questions.

Lower airway inflammation is a hallmark of patients with CF and increases with age and disease progression [28]; however, the host immune response is not uniform toward all microbes. For example, some viral infections of the lower respiratory tract (respiratory syncytial virus and influenza A, in particular) are associated with an even further elevated proinflammatory response and neutrophil influx [10]. Meanwhile, allergic bronchopulmonary aspergillosus, a clinical condition caused by immune sensitization to *Aspergillus fumigatus* antigens that develops in 4%–15% of patients with CF, is associated with enhanced type 2 T-helper cells [29]. These are just two examples within a breadth of differential host responses delivered against the complex polymicrobial infections in CF airways. Identification and determination of the molecular mechanism behind a multitude of different host responses engaged during stability versus exacerbation may provide novel biomarkers for monitoring patient infection status and guiding targeted treatment strategies.

The keys to optimal patient treatment are not clear. While we now recognize the importance of the polymicrobial nature of CF lung infections, we have a long way to go to understand how such communities, and the host’s response to those communities, contribute to disease. Conventional culture-based methods of diagnosing infections and determining antibiotic susceptibilities are expensive yet yield limited information about the composition and function of these polymicrobial infections, while drug susceptibility profiles provide minimal correlation with clinical outcome [30]. In the near future, embracing new diagnostic methods in the clinic, including utilization of rapidly advancing molecular-based methods, identifying biological markers of disease progression, establishing the impact of polymicrobial infection on host and treatment outcome, and tailoring treatment patient-by-patient will be essential in proactively treating and delaying clinical worsening.

## Conclusions

With the explosion of 16S rRNA gene deep sequencing studies performed over the past few years analyzing the microbial populations in the airways of CF patients, we face a new challenge: what does it mean? To begin to understand the impact of these polymicrobial infections on disease progression, we must study not only their composition but also their dynamics, the effects of inter-microbial and host—microbe interactions, the role of diverse host factors (e.g., genetics, immune response, and environment), and the impact of clinical intervention. Researchers are just beginning to tackle these issues. Most importantly, upon elucidation of complex, multifactorial disease mechanisms impacting CF lung infections, the ultimate challenge will be to use this information to develop new therapeutics, personalize care, and optimize treatment strategies.
